# Enhanced Oxidation of Carbamazepine Using Mn(II)-Activated Peracetic Acid: A Novel Advanced Oxidation Process Involving the Significant Role of Ligand Effects

**DOI:** 10.3390/molecules30132690

**Published:** 2025-06-21

**Authors:** Xue Yang, Hai Yu, Liang Hong, Zhihang Huang, Qinda Zeng, Xiao Yao, Yinyuan Qiu

**Affiliations:** 1 School of Ecological Environment and Urban Construction, Fujian University of Technology, Fuzhou 350118, China; 19046048r@connect.polyu.hk (X.Y.);; 2 School of Mechanical and Automotive Engineering, Fujian University of Technology, Fuzhou 350118, China; 3 Orbusneich Medical Shenzhen Co. Ltd., Shenzhen 518000, China; 4 Fujian Special Equipment Inspection and Research Institute, Fuzhou 350008, China

**Keywords:** peracetic acid, complexing ligands, high-valent Mn species, organic contaminants, wastewater treatment

## Abstract

In recent years, extensive attention has been paid to advanced oxidation processes (AOPs) with peracetic acid (PAA), a widely used disinfectant, using transition metal ions for the degradation of organic contaminants within water environments. Mn(II) has been widely used as an effective homogeneous transition metal catalyst for oxidant activation, but it has shown poor performances with PAA. Since the stability of manganese species can be enhanced through the addition of ligands, this study systematically investigated a novel AOP for the oxidation of carbamazepine (CBZ) using an Mn(II)/PAA system with several different ligands added. The reactive species were explored through UV-vis spectrometry, scavengers, and probe compounds. The results suggest that Mn(III)–ligand complexes and other high-valent Mn species (Mn(V)) were generated and contributed obviously toward efficient CBZ oxidation, while radicals like CH_3_CO_2_^•^ and CH_3_CO_3_^•^ were minor contributors. The oxidation efficiency of Mn(II)/PAA/ligands depended highly on ligand species, as ethylene diamine tetraacetic acid (EDTA) and oxalate (SO) could promote the oxidation of CBZ, while pyrophosphate (PPP) showed modest enhancement. The results obtained here might contribute to the removal of residue pharmaceuticals under manganese-rich waters and also shed light on PAA-based AOPs that could help broaden our present knowledge of manganese chemistry for decontamination in water treatment.

## 1. Introduction

With the development of urbanization and industrialization, attention to environmental issues concerning aquatic ecosystems has increased attention due to the growing number of emerging contaminants, i.e., pharmaceuticals, personal care products, endocrine disruptors, artificial sweeteners, disinfection by-products, and pesticides, which are continuously introduced into waterbodies worldwide [1]. Thus, advanced oxidation processes (AOPs) are promoted for the elimination of these recalcitrant contaminants in water and have attracted extensive attention due to their special advantages, including higher mineralization efficiency and mild operating conditions. The main principle of AOPs is the generation of reactive oxidation radicals (ROS) or reactive species with high redox potential from oxidants through activators like catalysts, UV, and heat. Specifically, peracetic acid (PAA) is an organic peroxyacid originally used in, for example, disinfectants, sanitizers, bleach, food industry, oxidizers, and polymerization catalysts, and unlike common oxidants like H_2_O_2_, peroxymonosulfate (PMS), and persulfate (PS), it has been confirmed to be effective in generating ROS with high reaction activity and thus able to degrade organic contaminants like diclofenac (DFC) and sulfamethexazole (SMX) through PAA-based AOPs [2,3,4,5].

Compared with some conventional water disinfectants and microbial inactivation, such as through chlorination, ozonation, and ultraviolet (UV), PAA is advantageous due to its high sterilization ability, lower pH dependence, easier technical implementation, and reduced formation of toxic by-products in the treated effluent [1,6,7]. In addition to its use as a disinfectant, it has also been reported that PAA has the potential for degrading aqueous organic contaminants, such as pharmaceuticals, due to its high oxidation potential, which ranges between 1.06 and 1.96 based on the working pH [8,9]. However, the application of PAA in AOPs for the removal of organic contaminants from water has received much less attention to date.

Generally, PAA can be activated using, for example, external energy like radiation (i.e., solar radiation, UV radiation, and microwave) and catalysts to produce reactive species for the oxidation of organic pollutants like fluoroquinolone, sulfadiazine, and bisphenol-A [10,11,12,13,14]. Based on previous studies, PAA can be decomposed into reactive species including peroxyl radicals (CH_3_CO_2_^•^ and CH_3_CO_3_^•^) from PAA molecules and ^•^OH from either PAA or coexisting H_2_O_2_ [15,16].

In addition to some common transition metal ions such as Fe(II/III), Cu(II), Mn(II), and their divertive composites are widely used in catalysis fields due to their special characterizations like natural abundant and valence variation. Manganese (Mn) exists naturally on earth and in space in several oxidation states, including Mn(II), Mn(III), Mn(IV), and Mn(V). However, in water, only Mn(II) is stable as a free ion. Soluble forms of higher oxidation states—such as Mn(III), Mn(V), and Mn(VI)—are generally unstable in solution. In other words, these Mn species in higher oxidation states do not typically exist as free ions in water. Instead, they are either short-lived intermediates that can be stabilized through complexation with ligands, or they quickly convert into other Mn species. They tend to undergo disproportionation, meaning they break down into more stable forms like Mn(II), Mn(IV) (which often forms insoluble oxides), or Mn(VII) (permanganate) [17,18]. Among them, aqueous Mn(II) ion is one of the dominant species in natural water environments; it is often regarded as an efficient trigger or acceleration for the activation of several oxidants such as persulfates and H_2_O_2_ [19,20,21]. However, the homogeneous activation of PAA using soluble Mn(II) has seldom been investigated.

It has been reported that the addition of some complexing ligands into the AOP reaction system with transitional metal ions might pose potential influence on the oxidation process with accelerated organic contamination degradation. As reported by previous works, ligands like ethylene diamine tetraacetic acid (EDTA), nitrilotriacetic acid (NTA), and sodium tripolyphosphate (TPP) could help boost the working efficiency of some Fe(II)-based AOPs, i.e., dissolved Fe(II), pyrite, magnetite, and Fe(II)-bearing clay minerals, by increasing the yield of ^•^OH production. This is because ligands might help utilize the electronic properties of Fe(II), thereby improving its ability in AOPs for the degradation of organic contaminants. For example, Xie et al. reported that the induction of specific ligands with high electron donation ability could obviously promote the production of ^•^OH through the use of Fe(II) since the decreasing electron donation ability of critical ligands could coordinate with Fe^2+^. In other words, regulating Fe(II) species using ligands with slight electron donation ability through ion exchange could enhance electron utilization efficiency during sediment oxygenation [22]. Zeng et al. reported that the presence of ligands could promote the yield and rate of ^•^OH production with an accelerated electron transfer rate since ligands can dissolve a part of solid Fe(II) into liquid Fe(III) [23]. Furthermore, some studies have also reported that in spite of the enhanced reactive species generation, the production of some other high-valent metal species after the addition of electron-donating ligands might also promote the oxidation and degradation of organic pollutants through the production of reactive intermediates. These high-valent metal species can activate oxidants like PMS and H_2_O_2_ through non-radical means, possibly due to the unique redox properties of the centered metal atom, and also play a major role as an alternative to reactive species in the removal of organic pollutants [24,25]. For example, Wang et al. reported that the presence of electron-donating ligands, the 4-aminopyridine species, accelerated the degradation of Reactive Red 195 using activated H_2_O_2_ due to the formation of IDA/Cu(II)/4-ampy complex. The generated high-valent copper–oxo species, [^•^O-Cu^III^(IDA)(4-ampy)_2_], was proposed as the possible main working active species since ^•^OH was not detected during the reaction [26]. Li et al. reported that the complex of Fe^III^-TAML was not only capable of PMS activation but also acted as the key active species responsible for the elimination of p-chlorophenol within a wide pH range [27].

The introduction of specific complexing ligands has been confirmed to promote the working efficiency within several Fenton-like AOPs activated by transitional metal species, i.e., PMS and H_2_O_2_ activated by Fe or Mn species, for the degradation of several organic pollutants such as dyes and pharmaceuticals. However, whether similar effects would occur within an AOP using PAA has rarely been investigated. Thus, there still lies a knowledge gap regarding the effects of ligands within Mn(II)/PAA, the reaction mechanism behind it, the kinds of main working reactive species, and the possible influence of additive ligands on the oxidation of certain organic contaminants.

The influences from additive ligands were explored using representatives such as sodium oxalate (SO), ethylene diamine tetraacetic acid (EDTA), and pyrophosphate (PPP). EDTA is known as an important chemical raw material widely used in the industrial production of textiles and chemicals. It is also a suitable chelating agent for a Fenton-type process, since with an iron–EDTA complex, the effective reduction of Fe(III) to Fe(II) using hydrogen peroxide drastically improves the catalytic nature of iron at near-neutral pH [28]. PPP belongs to the group of inorganic phosphates commonly used in meat industries [29]. It can not only improve the degradation of trichloroethylene (TCE) at neutral pH as an iron-stabilizing agent but also works as a strong complexing ligand to sequester Fe(V) oxidation during chemical oxidation [30,31]. The acceleration performances of SO within both a heterogeneous Fenton-like system and ozonation using activated Ce(III/IV) for efficient water treatment have also been significant [32,33]. Compared to with Fe species, the impacts from ligands toward oxidations using Mn species have been less investigated.

Thus, in this work, we systematically aimed to investigate (1) the activation performance of PAA using Mn(II) for the oxidation of a specific pharmaceutical contaminant, (2) the effects and reaction mechanisms of the additive ligands, namely EDTA, SO, and PPP, on the oxidation efficiency of the Mn(II)/PAA/ligand system, and (3) the identification of the major reactive species’ generated and their contributions within the oxidation. Herein, carbamazepine (CBZ) with antiepileptic and psychotropic activities was chosen as a model contaminant due to its low sorption properties and high resistance to biodegradation. In addition, not only was CBZ detected within municipal sewage treatment plants and domestic wastewater, but it was also one of the most frequently detected pharmaceuticals in drinking water systems in Europe and the USA [34].

## 2. Results and Discussion

This section is divided into subsections providing a concise and precise description of the experimental results, their interpretation, and the experimental conclusions that can be drawn.

### 2.1. Effect of Complexing Ligands in the Activation of PAA Using Mn(II)

**Figure 1:**
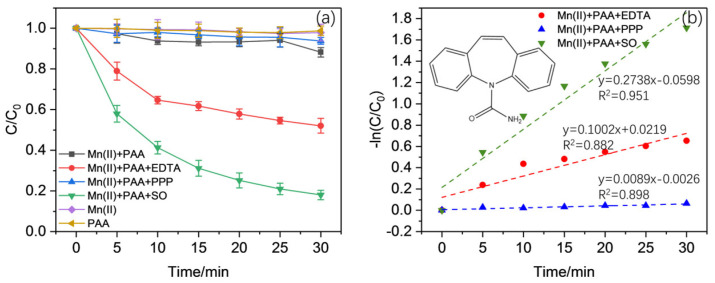
Oxidation of CBZ using Mn(II)/PAA/ligand system under different reaction conditions (**a**) and linear fit of Mn(II)/PAA/ligand systems (**b**).

The oxidation of CBZ with PAA using Mn(II) was investigated, and the results are provided in Figure 1. The results show that with no Mn(II) or PAA added, the concentration of CBZ remained almost unchanged during the whole reaction. The simultaneous presence of Mn(II) and PAA could barely help to change this situation, as residue CBZ decreased slightly after the reaction, with only about 12% removed. These situations reveal that PAA might hardly be activated by Mn^2+^ without further assistance.

(General working conditions: [Mn(II)] = 50 μM, [PAA] = 200 μM, [SO] = [EDTA] = [PPP] = 100 μM, pH = 5.5)

Thus, another series of experiments with three different ligands, namely EDTA, SO, and PPP, added into the Mn(II)/PAA system were performed. In order to determine the optimal working conditions for CBZ oxidation with ligands, the working parameters, including pH and the concentrations of Mn(II), ligands, and PAA, were investigated separately.

The effects of three ligands, namely EDTA, PPP, and SO, on the oxidation of CBZ are shown in Figure 1. The results imply that the three ligands posed significantly different impacts on the oxidation of CBZ, as EDTA and SO could promote CBZ oxidation, while PPP showed modest effects on the reaction. More than 80% and 50% of CBZ were removed within 30 min with the addition of SO or EDTA, separately. Furthermore, the oxidation trend of CBZ using SO and EDTA showed slight differences, as the one using SO fitted the pseudo-first-order model roughly, while the one using EDTA could be roughly divided into two stages: an initial rapid stage followed by a lagged second one.

**Figure 2:**
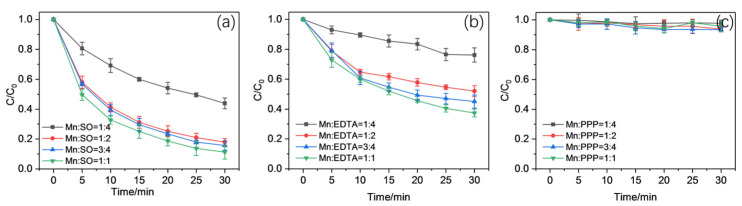
Effects of the ratio between Mn(II) and ligand concentration on the oxidation of CBZ in the (**a**) Mn(II)/PAA/SO system, (**b**) Mn(II)/PAA/EDTA system, and (**c**) Mn(II)/PAA/PPP system.

**Figure 3:**
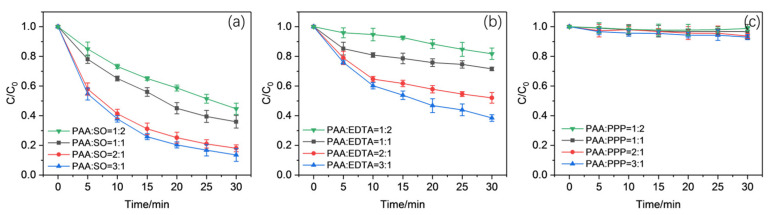
Effects of the ratio between PAA and ligand concentration on the oxidation of CBZ in the (**a**) Mn(II)/PAA/SO system, (**b**) Mn(II)/PAA/EDTA system, and (**c**) Mn(II)/PAA/PPP system.

As revealed in Figure 2, while increasing the Mn(II) concentration and keeping the ligands unchanged, the reduction of CBZ was promoted gradually, in general, with EDTA or SO added. This suggests a possible enhanced activation of PAA or the generation of some other oxidants with high redox potential using Mn(II) with co-existed EDTA or SO. The impacts caused by the change in the PAA concentration on CBZ oxidation are observed in Figure 3, showing a similar phenomenon but not as obvious as the previous ones. The above situations indicated the possible integration between PAA, Mn(II), and EDTA or SO, and PAA might not act as the only oxidant during the process. The roles of EDTA and SO might not be limited to the assistance of PAA activation. However, it should be noted that little promotion of CBZ oxidation (less than 5%) could be detected, while the ratios between Mn(II) and SO or EDTA increased beyond 1:2, except for one at 1:4 (about 17% of CBZ remained after oxidation in the 1:1 group, about 20% in the 3:4 group, and about 22% in the 1:2 group). This might be caused by the limited amount of ligands or Mn(II) added. Based on the above situations, it could be inferred that the combination of Mn(II) and SO or EDTA played an important role upon CBZ oxidation, and the decomposition of PAA was less significant in comparison.

(Working conditions: [Mn(II)] = 25, 50, 75, 100 μM, [PAA] = 200 μM, [SO] = [EDTA] = [PPP] = 100 μΜ, pH = 5.5)

(Working conditions: [Mn(II)] = 50 μM, [PAA] = 50, 100, 200, 300 μM, [SO] = [EDTA] = [PPP] = 100 μΜ, pH = 5.5)

The characteristics of the ligands might also interfere with the oxidation of PAA due to their interactions with Mn(II). As a complex molecule containing multiple coordinating atoms, EDTA with six coordination sites (four O-donors from the carboxyl groups and two N-donors from amino groups) could form stable five-membered chelate rings with Mn(II), which accelerated effective PAA activation. In addition, the tetra-negative anion originated from the N-donors and O-donors of EDTA might create a tetra-negative anion that attracts Mn(II) [35]. The retarded oxidation of CBZ with PPP, a tetradentate ligand, might be caused by its fewer coordinating sites, with only four. Thus, more PPP is needed to chelate Mn(II). In addition, the more rigid and bulky structure of PPP also made it less effective at forming tight complexes with Mn(II) [36,37]. SO was the simplest dicarboxylic acid with lower molecular weight, significant acidity, and chelating ability. Relevant studies have also reported that oxalate radicals might react with dissolved oxygen within water to form radicals like HO_2_^•^and O_2_^•−^, which could further disproportionate to form H_2_O_2_ [38,39]. In addition, it has been reported that the reaction rate between oxalate or its dissociated species and ^•^OH is relatively slow. The slight ^•^OH scavenging rate of oxalates also implies it has little competition with the target contaminant.

**Figure 4:**
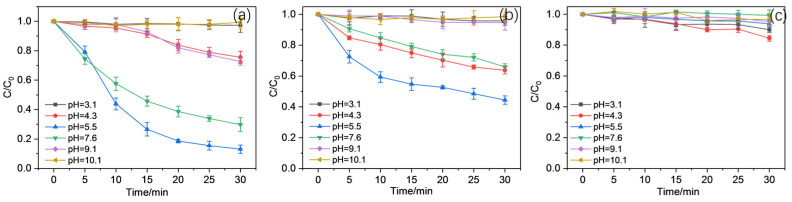
Effects of working pH on the oxidation of CBZ in the (**a**) Mn(II)/PAA/SO system, (**b**) Mn(II)/PAA/EDTA system, and (**c**) Mn(II)/PAA/PPP system.

The influence of working pH was also detected within the range of 3.0–10.0 to further investigate the optimal working condition for CBZ oxidation using Mn(II)/PAA/ligands, as indicated in Figure 4. Generally, more than 88% of CBZ oxidized could be found at pH 5.5 using SO, while little CBZ was removed in extreme acidic or alkaline environments. However, it was clear that the oxidation of CBZ with either SO or EDTA added was faster in the first 10 min at pH = 5.5 and was retarded obviously afterward. These phenomena might be caused by the speciation of PAA, as the pKa of PAA was ~8.2, indicating the predominant role of neutral species within the range of pH = 3–7. At the same time, at pH over 8.2, PAA mainly existed as disassociated species, PAA^−^, with lower oxidation ability than the protonated PAA near-neutral pH [40,41]. This specific situation helps explain the significantly inhibited CBZ oxidation in basic environments. Thus, a near-neutral working pH was more advantageous for the Mn(II)/PAA/ligand system.

(General working conditions: [Mn(II)] = 50 μM, [PAA] = 200 μM, [SO] = [EDTA] = [PPP] = 100 μΜ)

**Figure 5:**
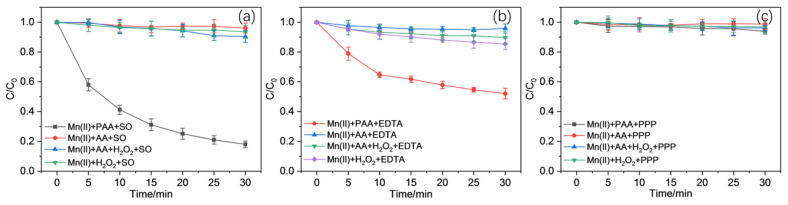
Oxidation of CBZ under different control conditions within (**a**) Mn(II)/SO, (**b**) Mn(II)/EDTA, and (**c**) Mn(II)/PPP systems.

In order to further investigate the important role PAA played during CBZ oxidation, a series of control experiments were performed using the raw materials for PAA synthesis, in other words, H_2_O_2_ and acetic acid (AA, CH_3_COOH). In addition, H_2_O_2_ and CH_3_COOH were also directly mixed without the addition of H_2_SO_4_, and the results are presented in Figure 5. It is obvious that none of H_2_O_2_, CH_3_COOH, or CH_3_COOH + H_2_O_2_ led to the oxidation of CBZ with Mn(II) added for homogeneous catalyis since the concentration of CBZ remained almost unchanged after the reaction. It has been widely reported that H_2_O_2_ could be effectively activated using transition metal ions or their oxides with the generation of ^•^OH, a common reactive species with high redox potential at around 2.8V [42]. However, the situation of H_2_O_2_ catalyzed with Mn(II) seemed no better than the others, which is consistent with previous studies in which H_2_O_2_ could hardly be activated using only Mn(II) [43]. Therefore, it is reasonable to conclude that PAA, instead of other reagents, was the main oxidant that could be activated with Mn(II) for the oxidation of CBZ.

(General working conditions: [Mn(II)] = 50 μM, [PAA] = 200 μM, [AA] = [H_2_O_2_] = 200 μM [SO] = [EDTA] = [PPP] = 100 μΜ, pH = 5.5)

### 2.2. Generation and Involvement of Mn(III) Complex

The above results indicate that the addition of specific ligands posed obvious interference on CBZ oxidation due to their impacts on PAA activation.

During the reaction between PAA and Mn(II), the disproportionation of Mn(III) was feasible to produce and stabilize with ligands or under acidic environments in situ, which helped prevent its dismutation into Mn(II) and Mn(IV) [36,44]. A similar situation was also detected within AOPs using oxidants like H_2_O_2_ and PMS, where the produced Mn(III) might turn into more stabilized Mn(III) species (e.g., Mn(III)-EDTA, Mn(III)-EDDS, and Mn(III)-ligand complexes with humic acid), demonstrating an effective reactive intermediate that contributes to the oxidation of organic contaminants like p-nitrophenol through carboxyl oxygen atoms, chlorophene, and atrazine [36,45,46,47].

**Figure 6:**
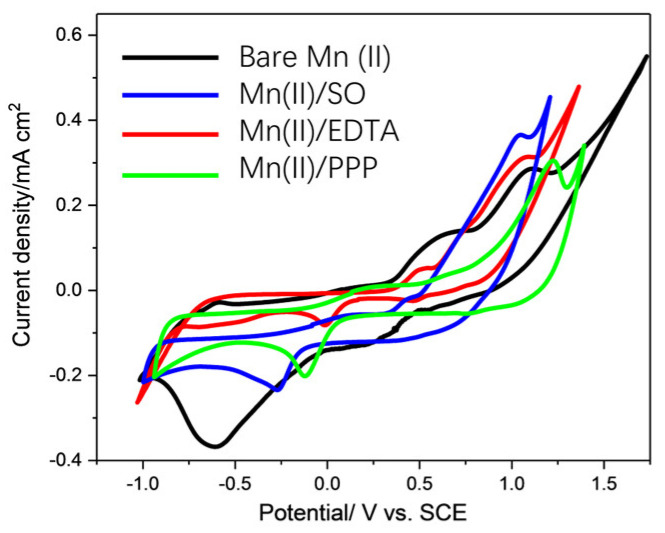
Cyclic voltammetry (CV) profiles of bare Mn(II), Mn(II)/SO, Mn(II)/EDTA, and Mn(II) PPP.

In order to further clarify the changes in Mn(II)/Mn(III) potential in the presence of the three different chelating agents, a cyclic voltammetry test was performed (Figure 6). The cyclic voltammetry (CV) curve of bare Mn^2+^ solution showed two oxidation peaks located at around 0.68 and 0.99 V, which could be attributed to the transformation of Mn(II) to Mn(III) and of Mn(III) to Mn(IV) on the electrode surface, respectively [48,49]. The addition of SO significantly promoted the conversion of Mn(II) to Mn(III) by lowering the oxidation potential of Mn(II)/Mn(III) (ΔE ≈ −0.22 V) and enhanced the peak current. These findings reveal the coordination of the accelerated electron transfer process, where the strong chelating ability of SO contributes to the stabilization of the Mn(III) species and thus reduces the activation energy barrier [50,51,52].

A similar phenomenon was also found with the addition of EDTA, but with only a slight shift in the oxidation potential (ΔE ≈ −0.15 V). These effects might be caused by its stronger chelation strength (logK = 13.6) compared to that of SO (logK = 5.2), leading to the inhibitory behavior caused by the over-coordination observed in stronger ligand systems [51,53]. Herein, it could be concluded that the addition of SO showed higher tendency to stabilize Mn(III) species [52]. However, the peak current with the addition of PPP reduced significantly, which might result from the suppression of electron transfer. In addition, the oxidation potential shifted toward higher potential, which indicated a stronger activation energy barrier [54]. The ligand environment in Mn-based catalysts might critically regulate intermediate stability during hydrogenation reactions. It has been reported that PPP is likely to coordinate with Mn(II) to form more stable complexes, thereby reducing its propensity to oxidize into highly reactive Mn(III/IV) species [55]. In addition, PPP might also induce the formation of Mn(II)-P_2_O_7_ precipitates at near-neutral pH, thereby further reducing the concentration of active manganese species [56].

(General working conditions: [Mn(II)] = 50 μM, [PAA] = 200 μM, [SO] = [EDTA] = [PPP] = 100 μΜ, pH = 5.5)

**Figure 7:**
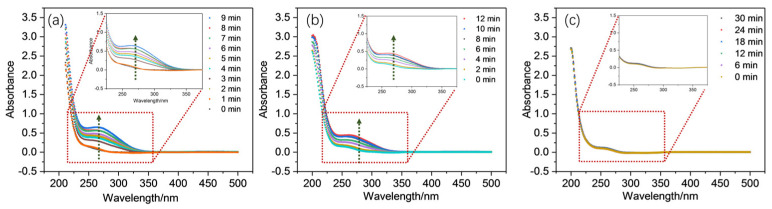
UV-vis spectra changes as the reactions of (**a**) Mn(II)/PAA/SO, (**b**) Mn(II)/PAA/EDTA, and (**c**) Mn(II)/PAA/PPP progress.

The presence of Mn(III) species within the PAA activation system could be determined using UV-vis spectra within the range of 200–500 nm since Mn(III) species have a characteristic adsorption peak at around 270 nm [45,57,58]. Figure 7 implies that the signals attributed to Mn(III) species were detected in both EDTA and SO systems minutes after the addition of PAA but with different strengths and formation rates. Generally, the strength of Mn(III) species’ signal increased dramatically in the first 4 min and slowed down gradually afterward. The electron transfer from both the outer sphere and intramolecular electron transfer from EDTA to Mn(III) helped make it more stable [59]. Compared to EDTA, Mn(III) appeared earlier with SO addition, indicating the possible acceleration of Mn(III) formation caused by SO. In contrast, there was almost no Mn(III) signal detected in the PPP system, revealing that Mn(III) could hardly be stabilized with PPP. The above findings imply the feasibility of oxidizing Mn(II) into Mn(III) species within PAA environments and that the complexing ligands play critical roles during this process. The different impacts on the oxidation of CBZ could be explained by the specific characterizations within the ligands. The Mn(III) species could hardly be formed through PPP, a non-redox active compound, which might be caused due to ligand hydrolysis and disproportionation [59,60]. Therefore, it is reasonable to infer that stable Mn(III) species might be more difficult to generate within a Mn(II)/PAA/PPP system than the one using EDTA.

As for SO, it could coordinate with Mn(II) through carboxyl oxygen atoms. The oxalate tended to bind to Mn(II) in a bidentate mode via both deprotonated oxygen atoms (O^−^) of the carboxylate groups, forming a stable five-membered chelate ring [61,62]. In this reaction system, such coordination helped to stabilize Mn(III), thus facilitating further oxidations [63,64]. However, the formation of Mn(III) might undergo disproportionation with the production of Mn(II) and MnO_2_, which showed less reactivity, as indicated by Equation (1). Herein, the presence of SO could help to stabilize Mn(III) species through the generation of Mn(III)–ligand complexes [65].

2Mn(III) + 2H_2_O → Mn(II) + MnO_2_ + 4H^+^ (1)

In addition, the simpler structure of SO showed lower steric hindrance than EDTA and PPP [66,67]. This might be conducive to the oxidation process with a more stable complex generated. Therefore, it is reasonable to infer that the generation and participation of Mn(III) species during the oxidation of CBZ using Mn(II)/PAA/ligands play critical roles.

### 2.3. Identification of ROS

The above results reveal that the oxidation of CBZ under Mn(II)/PAA/ligands was possibly due to the generation of reactive species from both PAA and Mn(III) species. Thus, a series of scavenging tests were performed using scavengers, including MeOH (methanol) and TBA (tertiary butanol). TBA is a highly reactive quencher toward ^•^OH (k_•OH/TBA_ = 3.8−7.6 × 10^8^ M^−1^·s^−1^), while MeOH can quench ^•^OH and ROH^•^ spontaneously (k_•OH/MeOH_ = 9.7 × 10^8^ M^−1^·s^−1^) [68,69].

(General working conditions: [Mn(II)] = 50 μM, [PAA] = 200 μM, [SO] = [EDTA] = [PPP] = 100 μΜ, pH = 5.5, [TBA] = [MeOH] = 50 mM, [PMSO] = 5 mM)

**Figure 8:**
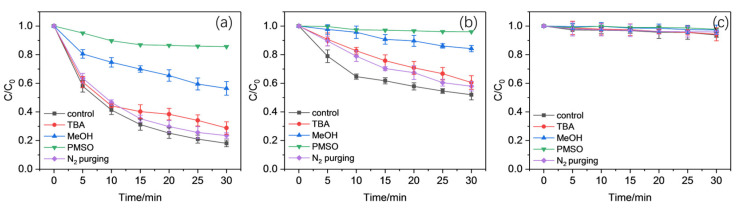
Effects from scavengers and N_2_ purging on the oxidation of CBZ in the (**a**) Mn(II)/PAA/SO, (**b**) Mn(II)/PAA/EDTA, and (**c**) Mn(II)/PAA/PPP systems.

Figure 8 reveals that although the addition of excessive TBA retarded the CBZ oxidation to a certain extent, the inhibition was slight, as residual CBZ only increased within 10% compared to the normal ones. The reaction was never terminated in the ones using SO and EDTA, indicating that ^•^OH was not the only effective reactive species involved in the oxidation of CBZ. The situation for the PPP group remained unchanged, with almost no CBZ oxidized.

The results in Figure 8 show far more obvious inhibition in CBZ oxidation with 50 mM MeOH addition compared to that with TBA, implying that the roles of ROH^•^ (i.e., CH_3_CO_2_^•^, CH_3_CO_3_^•^, CH_3_^•^, and CH_3_O_2_^•^) played a more critical role in the reaction. In addition, the recombination of CH_3_^•^ and PAA with dissolved O_2_ (DO) within the reaction system accelerated the formation of another peroxy radical, CH_3_O_2_^•^, which might also promote oxidation [70]. The anaerobic experiments under N_2_ plugging in Figure 8 showed that the oxidation of CBZ was partly interfered with compared to the normal ones, with a modest reduction in CBZ oxidation. This indicates that CH_3_O_2_^•^ might only contribute to the oxidation process slightly.

**Figure 9:**
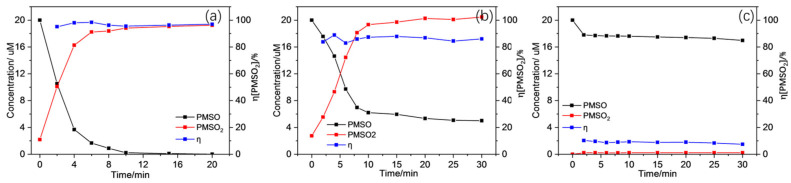
Conversion of PMSO and the yield of PMSO_2_ using (**a**) Mn(II)/PAA/SO, (**b**) Mn(II)/PAA/EDTA, and (**c**) Mn(II)/PAA/PPP).

Furthermore, since some high-valent Mn species (i.e., Mn(IV) and Mn(V) species) with high reactivity could be formed within the Mn(II)/PAA/ligand system using ligands like PICA, HA, and EDDS, some other investigations needed to be performed to distinguish their contributions from the others [36,44,71]. Herein, PMSO (methyl phenyl sulfoxide) was adopted as a mechanic probe to confirm the existence and contribution of high-valent Mn species like Mn(V). It has been reported that PMSO can hardly react with PAA and its characteristic reactive species, including CH_3_CO_2_^•^ and CH_3_CO_3_^•^, but can be converted to PMSO_2_ by high-valent metal species through oxygen atom transfer [72,73]. The products oxidized by free radicals like SO_4_^•−^ and ^•^OH were generally hydroxylated and/or polymeric products. Thus, the existence and contribution of high-valent Mn species could be determined using the generation rate and yield of PMSO_2_ (i.e., the molar ratio of produced PMSO_2_ to converted PMSO, η (PMSO_2_)). The results in Figure 9 reveal that the conversion of PMSO into PMSO_2_ using Mn(II)/PAA/ligands varied significantly over time at pH = 5.5. Generally, the addition of SO accelerated the conversion of PMSO into PMSO_2_ significantly with an obvious conversion yield, about 100%, in less than 5 min at most. Compared to SO, the conversion of PMSO with EDTA was slightly slower, with less than 85% converted into PMSO_2_ in about 10 min. As for PPP, almost no PMSO_2_ was generated. These situations indicate that the Mn(III) formed initially due to the reaction of Mn(II)-SO and Mn(II)-EDTA, with PAA showing a higher conversion rate in high-valent Mn species (Mn(V)) later than with PPP.

(General working conditions: [Mn(II)] = 50 μM, [PAA] = 200 μM, [SO] = [EDTA] = [PPP] = 100 μΜ, [PMSO] = 20 μM, pH = 5.5)

Based on the above results, it is clear that several reactive species and Mn species with different valences can be generated with the Mn(II)/PAA/ligand system for the oxidation of CBZ. However, MeOH could also quench the Mn(III) species or any high-valent Mn species generated in addition to CH_3_CO_2_^•^, CH_3_CO_3_^•^, and ^•^OH. Since traditional quenching experiments could hardly distinguish the relative contributions of CH_3_CO_2_^•^, CH_3_CO_3_^•^, and high-valent Mn species (Mn(V)) within a Mn(II)/PAA/ligand system, further investigations were performed. Herein, excessive PMSO was added into the reaction system as a scavenger, as indicated in Figure 9. The results show that the addition of PMSO inhibited CBZ oxidation using SO and EDTA seriously, where almost no CBZ could be removed throughout the reaction. This situation reveals the importance of high-valent Mn species (Mn(V)) in the oxidation of CBZ, while the contribution of CH_3_CO_2_^•^ and CH_3_CO_3_^•^ were slight.

Generally, both the traditional quenching experiments and PMSO oxidations helped in the determination of the main working reactive species during the Mn(II)/PAA/ligand process in the oxidation of CBZ. Except for PPP, the experiments using SO and EDTA showed that in addition to major ROS, including CH_3_CO_2_^•^ and CH_3_CO_3_^•^, Mn(III) species and high-valent Mn(V) also exhibited unignorably impacts. The limited influence from TBA and obvious inhibition from PMSO indicated the participation of Mn(III) and high-valent Mn(V) species within CBZ oxidation when SO and EDTA were added. However, the high conversion rate of PMSO into PMSO_2_ and the retardation from excessive PMSO on CBZ oxidation indicated the more dominant role of high-valent Mn(V) species compared to the others. Therefore, it could be concluded that Mn(III) species like Mn(III)-SO and Mn(III)-EDTA could be formed during the activation of PAA using Mn(II) with corresponding ligands added, while PPP would hardly facilitate the oxidation of Mn(II) into Mn(III) species. Furthermore, high-valent Mn(V) species could also be further formed with appropriate ligands like SO and EDTA within the Mn(II)/PAA/ligand system, which contributed largely to the oxidation of CBZ.

Thus, the possible reaction mechanism of Mn(II)/PAA/SO and Mn(II)/PAA/EDTA could be expressed using Equations (2)–(9) below. The coordination between Mn(II) and the ligands (SO or EDTA) led to the generation of Mn(II)-SO or Mn(II)-EDTA complexes. Then, the electron transfer between the Mn(II)-SO or Mn(II)-EDTA complex and PAA resulted in the formation of reactive species like CH_3_C(O)O^•^ and Mn(III) species. Afterward, the Mn(III) species would further react with PAA to produce high-valent Mn(V) species and/or CH_3_C(O)O^•^. These generated reactive species, including organic radicals (CH_3_C(O)O^•^ and CH_3_C(O)OO^•^) and Mn(V), contributed to the oxidation of CBZ [65,71].

For the Mn(II)/PAA/SO system:

Mn(II) + SO → Mn(II) − SO(2)

Mn(II) − SO + CH_3_C(O)OOH → Mn(III) − SO + CH_3_C(O)O^•^ + OH^−^ (3)

Mn(III) − SO + CH_3_C(O)OOH → Mn(V) − SO + CH_3_C(O)O^•^ + OH^−^ (4)

Mn(III) − SO + CH_3_C(O)OOH → Mn(II) − SO + CH_3_C(O)OO^•^ + H^+^ (5)

For the Mn(II)/PAA/EDTA system:

Mn(II) + EDTA → Mn(II) − EDTA(6)

Mn(II) − EDTA + CH_3_C(O)OOH → Mn(III) − EDTA + CH_3_C(O)O^•^ + OH^−^ (7)

Mn(III) − EDTA + CH3C(O)OOH → Mn(V) − EDTA + CH_3_C(O)O^•^ + OH^−^ (8)

Mn(III) − EDTA + CH3C(O)OOH → Mn(II) − EDTA + CH_3_C(O)OO^•^ + H^+^ (9)

### 2.4. Proposed Oxidation Pathway of CBZ

**Figure 10:**
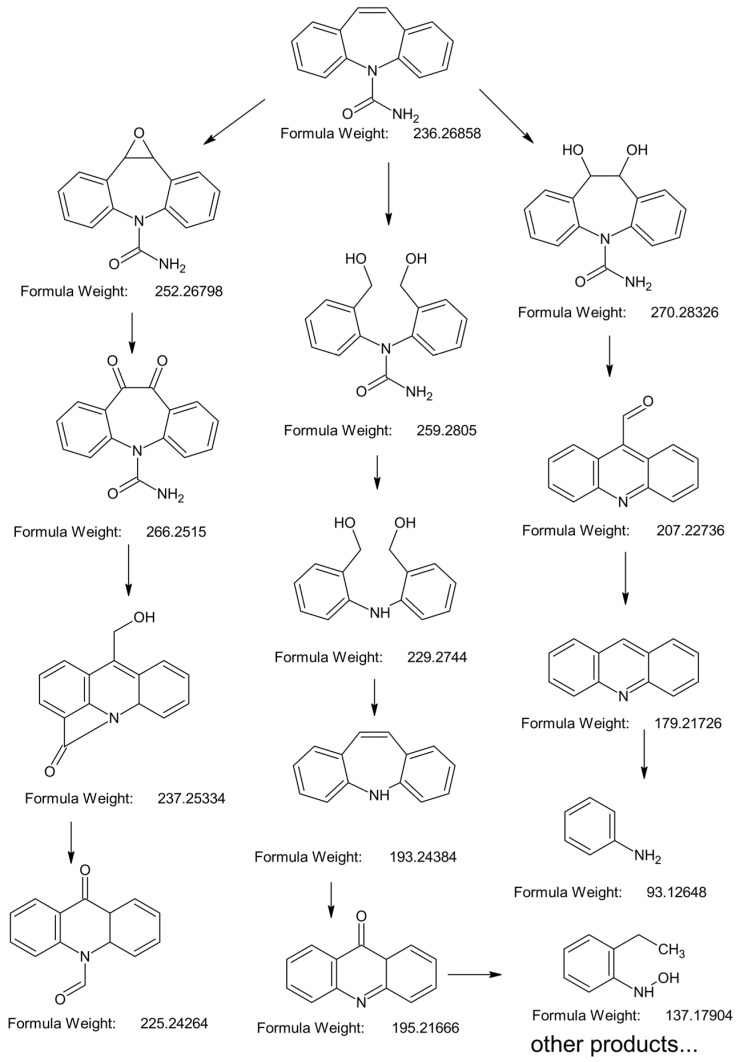
Proposed CBZ oxidation pathway within Mn(II)/PAA/SO system.

Generally, three oxidation pathways could be identified while the electrophilic attack on the olefinic double bond by reactive species initiated the oxidation (Figure 10). Subsequently, the following intermediates could be formed through subsequent reactions such as deacetylation, ring contraction, dehydrogenation, oxygen transfer, and dehydration, during which their structure was gradually simplified and their molar weight decreased. Among them, the electron-rich groups like C=C and C-N tended to be attacked by ^•^OH and high-valent Mn species, while the hydroxylation products might have resulted from the attack of ^•^OH with compounds containing electron-donating groups (such as –NH_2_) [63,74,75].

(General working conditions: [Mn(II)] = 50 μM, [PAA] = 200 μM, [SO] = 100 μΜ, pH = 5.5)

## 3. Materials and Methods

### 3.1. Chemical Reagents

Except for PAA, the other chemical reagents in this work were all of analytical purity or over and were used directly as obtained without further purification. The chemical reagents, including acetic acid (AA), 30% hydrogen peroxide (H_2_O_2_), MnSO_4_·H_2_O (Mn(II)), sodium oxalate (SO), sodium pyrophosphate (PPP), and sodium ethylene diamine tetraacetic acid (EDTA), were purchased from Macklin. Methyl phenyl sulfoxide (PMSO), methyl phenyl sulfone (PMSO_2_), tert-butyl-alcohol (TBA), and carbamazepine (CBZ) were obtained from Sigma-Aldrich. Acetonitrile (ACN) and methanol (MeOH) were purchased from Merck. All solutions were prepared using Milliq water (>18.2 MΩ·cm) from a Millipore system (Millipore, Billerica, MA, USA).

### 3.2. Synthesis of PAA

The PAA solution used in this work was prepared with acetic acid (AA) and 30% hydrogen peroxide (H_2_O_2_) under the catalysis of 98% H_2_SO_4_ in liquid phase at room temperature (25 °C), as stated by Equation (10).

(10) $${\mathrm{CH}}_{3}\mathrm{COOH}+H_{2}O_{2}\overset{H_{2}{\mathrm{SO}}_{4}}{\to }{\mathrm{CH}}_{3}\mathrm{COOOH}+H_{2}O$$

Briefly, 60 mL AA and 40 mL H_2_O_2_ were mixed in a 125 mL amber glass reagent bottle with a frosted mouth and cover, and 1 mL H_2_SO_4_ was added as the catalyst. Then, this airtight bottle was then placed under continuous magnetic stirring for 20 h at a speed of 1200 r/min to make the reaction fully processed [76,77]. All syntheses were performed in a fuming hood since AA, H_2_O_2_, and PAA were all volatile. The concentration of the obtained PAA after 20 h synthesis was determined via iodometric titration [7]. The PAA concentration of the synthesized solution was about 2.3 mol/L fresh and decreased by about 5% after 7 days according to our testing. Therefore, PAA was synthesized every 3 days to keep the freshness and a relatively accurate concentration.

### 3.3. Batch Experiments

CBZ was oxidized in a 50 mL amber iodine measuring flask with a frosted mouth and cover under continuous magnetic stirring in a fuming hood. Batch experiments were initiated by adding a predetermined amount of PAA into the mixed solution, which already contained the target compound, Mn(II), and ligands. The Mn(II) for the reaction came from the MnSO_4_·H_2_O stock solution. The dosages of PAA, Mn(II), and ligands varied according to the experiments and are stated in detail in the figure captions. The initial pH of the reactions was adjusted by H_2_SO_4_ (0.1 mol/L) and NaOH (0.1 mol/L). At given time intervals, 0.5 mL of the working solutions was withdrawn using a syringe and quickly quenched with excessive Na_2_S_2_O_3_ (10 mM). In addition to the traditional control experiments, control experiments were performed using the raw materials of PAA synthesis, in other words, using H_2_O_2_, AA, and H_2_O_2_ + AA as oxidants.

Traditional quenching experiments were performed to determine the generation and participation of reactive species like ^•^OH, peroxyl radicals (CH_3_CO_2_^•^ and CH_3_CO_3_^•^), and high-valent Mn species. Herein, scavengers including tert-butyl alcohol (TBA), methanol (MeOH), and methyl phenyl sulfoxide (PMSO) were adopted.

All oxidation experiments were conducted thrice with relative standard deviations less than 5%.

### 3.4. Analytical Methods

The concentrations of CBZ, PMSO, and PMSO_2_ were analyzed using a High-Performance Liquid Chromatography (HPLC, Waters, MA, USA) system equipped with a DAD detector and a reverse-phase C18 column (Restek, 5 μm, 250 × 4.6 mm) at a flow rate of 1 mL/min under room temperature. The HPLC analyses for CBZ, PMSO, and PMSO_2_ were all performed using isocratic elution. The mobile phase for CBZ consisted of 40% of MilliQ water and 60% of ACN, with the detection wavelength set at 286 nm. The mobile phase for PMSO consisted of 50% of 0.1% (*v*/*v*) formic acid in water and 50% of ACN, with the detection wavelength set at 230 nm. The mobile phase for PMSO_2_ consisted of 50% of 0.1% (*v*/*v*) formic acid in water and 50% of ACN, with the detection wavelength set at 215 nm [78]. The oxidation intermediates of CBZ were identified using Liquid Chromatography coupled with an Orbitrap Mass Spectrometer (LC-MS, Thermo Scientific, MA, USA) with positive-mode electrospray ionization (ESI^+^). Chromatographic separation was carried out on a C18 HPLC capillary column (5 μm, 100 mm × 2.1 mm). The eluents consisted of deionized water (eluent A, containing 0.1% formic acid) and ACN (eluent B) with a flow rate of 0.25 mL min^−1^. The gradient program was started at 95% A for 12 min, followed by a decrement to 5% A in 5 min and then went back to 95% A in 5 min. The column temperature and injection volume were set at 30 °C and 10 μL. The cyclic voltammetry (CV) was performed with a Chenhua electrochemical workstation in a three-electrode electrochemical cell comprising a Pt wire counter electrode, a saturated Ag/AgCl reference electrode, and a glassy carbon electrode as the working electrode at room temperature. The voltammetry results were collected at a potential scan rate of 10 mV/s. Finally, the UV-vis spectra were scanned using a Shimadzu UV2600i spectrometer (Shimadzu, Japan).

## 4. Conclusions

This study mainly focused on the efficient oxidation of CBZ through the activation of PAA, a sufficient disinfectant traditionally used in water treatment, in the textile industry, and in the sterilization of equipment in healthcare settings. As PAA is difficult to activate using solely Mn(II), this work focused on the enhanced oxidation efficiency of a Mn(II)/PAA system through the addition of chelated ligands. The optimal working parameters, such as the working pH, dosages of PAA, and ratio between Mn(II) and ligands, were investigated separately. The results show that SO and EDTA could significantly promote oxidation efficiency at near-neutral pH, while PPP only slightly influenced the reactions. Mn(III) species could be stabilized effectively using appropriate ligands like SO and EDTA, and later investigations showed that these stabilized Mn (III) species could even be further generated into high-valent Mn species (Mn(V)), which also contributed obviously to the oxidation of CBZ in addition to CH_3_CO_2_^•^ and CH_3_CO_3_^•^. This study sheds light on a new advanced oxidation process, which helps broaden present knowledge of PAA activation as well as manganese chemistry for decontamination in water treatment.

## Data Availability

The data presented in this study are available on request from the corresponding author.

## References

[1] Eloranta J., Li C., Ao X.-W., Lu Z.-D., Huang C.-H., Santoro D., Sun W.-J. (2021). Peracetic acid-based advanced oxidation processes for decontamination and disinfection of water: A review. Water Res..

[2] Deng S., Yang Z., Yu X., Li M., Cao H. (2024). The reactivity of organic radicals in the performic, peracetic, perpropionic acids-based advanced oxidation process: A case study of sulfamethoxazole. J. Hazard. Mater..

[3] Correa-Sanchez S., Peñuela G.A. (2022). Peracetic acid-based advanced oxidation processes for the degradation of emerging pollutants: A critical review. J. Water Process. Eng..

[4] Chen S.H., Fegan N., Kocharunchitt C., Bowman J.P., Duffy L.L. (2020). Effect of peracetic acid on Campylobacter in food matrices mimicking commercial poultry processing. Food Control.

[5] Lin J., Zou J., Cai H., Huang Y., Li J., Xiao J., Yuan B., Ma J. (2021). Hydroxylamine enhanced Fe (II)-activated peracetic acid process for diclofenac degradation: Efficiency, mechanism and effects of various parameters. Water Res..

[6] Koivunen J., Heinonen-Tanski H. (2005). Peracetic acid (PAA) disinfection of primary, secondary and tertiary treated municipal wastewaters. Water Res..

[7] Cheng C., Li H., Wang J., Wang H., Yang X. (2020). A review of measurement methods for peracetic acid (PAA). Front. Environ. Sci. Eng..

[8] Popescu E.M., Pantea O., Gologan D., Doukeh R. (2019). Hydrogen Peroxide and Peracetic Acid Oxidizing Potential in the Treatment of Water. Rev. Chim..

[9] Zhang C., Brown P.J., Hu Z. (2018). Thermodynamic properties of an emerging chemical disinfectant, peracetic acid. Sci. Total. Environ..

[10] Wang L., Wei J., Li Y., Huo J., Ji W., Cui N., Li J., Niu X., Jiang Z., Cui X. (2023). A state-of-the-art review on heterogeneous catalysts-mediated activation of peracetic acid for micropollutants degradation: Classification of reaction pathways, mechanisms, influencing factors and DFT calculation. Chem. Eng. J..

[11] Ao X., Zhang X., Li S., Yang Y., Sun W., Li Z. (2022). Comprehensive understanding of fluoroquinolone degradation via MPUV/PAA process: Radical chemistry, matrix effects, degradation pathways, and toxicity. J. Hazard. Mater..

[12] Gao Y., Liu B., Huang X., Ma Y., Wang H. (2025). Insight into Three-Dimensional Electro-Fenton system with Fe0 activated Peroxyacetic acid for sulfadiazine degradation under neutral Condition: Performance and degradation pathways. Chem. Eng. J..

[13] Yang S.-R., He C.-S., Xie Z.-H., Li L.-L., Xiong Z.-K., Zhang H., Zhou P., Jiang F., Mu Y., Lai B. (2022). Efficient activation of PAA by FeS for fast removal of pharmaceuticals: The dual role of sulfur species in regulating the reactive oxidized species. Water Res..

[14] Shao Y., Li S., Wei X., Zhao Y., Liang J., Li X. (2023). The diverse roles of halide ions in the degradation of bisphenol A via UV/peracetic acid process at different pH values: Radical chemistry, and transformation pathways. J. Hazard. Mater..

[15] Chen S., Cai M., Liu Y., Zhang L., Feng L. (2019). Effects of water matrices on the degradation of naproxen by reactive radicals in the UV/peracetic acid process. Water Res..

[16] Huang C.-H., Zhang T. (2020). Modeling the kinetics of UV/peracetic acid advanced oxidation process. Environ. Sci. Technol..

[17] Nakamura R. (2013). (Invited) Design Principle of Multi-Electron Water Oxidation Catalysts Composed of Mn Oxides. ECS Meet. Abstr..

[18] Medina E.A., Li J., Stalick J.K., Subramanian M. (2016). Intense turquoise colors of apatite-type compounds with Mn^5+^ in tetrahedral coordination. Solid State Sci..

[19] Morgan J.J. (2000). Manganese in natural waters and earth’s crust: Its availability to organisms. Met. Ions Biol. Syst..

[20] Yang Z., Pan B., Shan C., Pignatello J.J. (2022). Mn(II) acceleration of the picolinic acid-assisted Fenton reaction: New insight into the role of manganese in homogeneous Fenton AOPs. Environ. Sci. Technol..

[21] Zhou Q., Wang Q., Tong S. (2017). Mn^2+^/H_2_O_2_/O_3_, a high efficient advanced oxidation process in acidic solution. J. Environ. Chem. Eng..

[22] Zhang P., Yuan S., Liao W., Tong M., Xie W. (2021). Ligand-Enhanced Electron Utilization for Trichloroethylene Degradation by ^·^OH during Sediment Oxygenation. Environ. Sci. Technol..

[23] Zeng Q., Dong H., Wang X. (2019). Effect of ligands on the production of oxidants from oxygenation of reduced Fe-bearing clay mineral nontronite. Geochim. Cosmochim. Acta.

[24] Pang S.-Y., Qiu W., Guo Q., Wang Z., Guan C., Jiang J. (2022). Aqueous iron (IV)–oxo complex: An emerging powerful reactive oxidant formed by iron (II)-based advanced oxidation processes for oxidative water treatment. Environ. Sci. Technol..

[25] Zong Y., Zhang H., Zhang X., Liu W., Xu L., Wu D. (2022). High-valent cobalt-oxo species triggers hydroxyl radical for collaborative environmental decontamination. Appl. Catal. B Environ..

[26] Wang Y., Xia H., Sun K., Wu S., Lu W., Xu J., Li N., Pei K., Zhu Z., Chen W. (2016). Insights into the generation of high-valent copper-oxo species in ligand-modulated catalytic system for oxidizing organic pollutants. Chem. Eng. J..

[27] Li H., Shan C., Li W., Pan B. (2018). Peroxymonosulfate activation by iron(III)-tetraamidomacrocyclic ligand for degradation of organic pollutants via high-valent iron-oxo complex. Water Res..

[28] Rastogi A., Al-Abed S.R., Dionysiou D.D. (2009). Effect of inorganic, synthetic and naturally occurring chelating agents on Fe(II) mediated advanced oxidation of chlorophenols. Water Res..

[29] Molina R.E., Bohrer B.M., Mejia S.M.V. (2023). Phosphate alternatives for meat processing and challenges for the industry: A critical review. Food Res. Int..

[30] Huang Z.-S., Wang L., Liu Y.-L., Zhang H.-Y., Zhao X.-N., Bai Y., Ma J. (2021). Ferrate self-decomposition in water is also a self-activation process: Role of Fe(V) species and enhancement with Fe(III) in methyl phenyl sulfoxide oxidation by excess ferrate. Water Res..

[31] Wang X., Brusseau M.L. (1998). Effect of pyrophosphate on the dechlorination of tetrachloroethene by the Fenton reaction. Environ. Toxicol. Chem..

[32] Xue X., Hanna K., Despas C., Wu F., Deng N. (2009). Effect of chelating agent on the oxidation rate of PCP in the magnetite/H_2_O_2_ system at neutral pH. J. Mol. Catal. A Chem..

[33] Zhang J., Shan C., Zhang W., Pan B. (2023). In situ ligand-modulated activation of inert Ce(III/IV) into ozonation catalyst for efficient water treatment. Proc. Natl. Acad. Sci. USA.

[34] Soufan M., Deborde M., Delmont A., Legube B. (2013). Aqueous chlorination of carbamazepine: Kinetic study and transformation product identification. Water Res..

[35] Zhang Y., Zhou M. (2019). A critical review of the application of chelating agents to enable Fenton and Fenton-like reactions at high pH values. J. Hazard. Mater..

[36] Zhao M., Huang D., Xiao J., Dong J., Li L., Dong H. (2024). Enhanced Degradation of Micropollutants in a Peracetic Acid/Mn(II) System with EDDS: An Investigation of the Role of Mn Species. Environ. Sci. Technol..

[37] Qiao J., Liu W., Guan X., Sun B. (2019). Influence of Pyrophosphate on the Generation of Soluble Mn(III) from Reactions Involving Mn Oxides and Mn(VII). Environ. Sci. Technol..

[38] Kwan C., Chu W. (2007). The role of organic ligands in ferrous-induced photochemical degradation of 2,4-dichlorophenoxyacetic acid. Chemosphere.

[39] Chen Q., Zhou M., Pan Y., Zhang Y. (2023). Ligand-Enhanced Zero-Valent Iron for Organic Contaminants Degradation: A Mini Review. Processes.

[40] Kiejza D., Kotowska U., Polińska W., Karpińska J. (2021). Peracids-New oxidants in advanced oxidation processes: The use of peracetic acid, peroxymonosulfate, and persulfate salts in the removal of organic micropollutants of emerging concern—A review. Sci. Total Environ..

[41] Kim J., Zhang T., Du P., Dobson J.T., Huang C.-H., Liu W. (2019). Advanced Oxidation Process with Peracetic Acid and Fe(II) for Contaminant Degradation. Environ. Sci. Technol..

[42] Tian C., Li J., Li Q., Nie Y., Tian X., Dai C., Yang C., Zhou Z., Wang Y. (2020). Surface weak acid-base pair of FeOOH/Al_2_O_3_ for enhanced peroxymonosulfate activation in degradation of humic substances from water. Chem. Eng. J..

[43] Ren Z.-H., Li H.-T., Xia K.-S., Gao Q., Han B., Zhou C.-G. (2017). Partial-Redox-Promoted Mn Cycling of Mn(II)-Doped Heterogeneous Catalyst for Efficient H_2_O_2_-Mediated Oxidation. ACS Appl. Mater. Interfaces.

[44] Kim J., Sharma V.K., Huang C.-H., Wang J., Ashley D.C. (2023). Picolinic Acid-Mediated Catalysis of Mn(II) for Peracetic Acid Oxidation Processes: Formation of High-Valent Mn Species. Environ. Sci. Technol..

[45] Gao Y., Zhou Y., Pang S.-Y., Jiang J., Shen Y.-M., Song Y., Duan J.-B., Guo Q. (2021). Enhanced peroxymonosulfate activation via complexed Mn(II): A novel non-radical oxidation mechanism involving manganese intermediates. Water Res..

[46] Ge J., Wang S., Gu C., Wang X., Qu R., Wang Z. (2018). Enhanced Removal of Chlorophene and 17β-estradiol by Mn(III) in a Mixture Solution with Humic Acid: Investigation of Reaction Kinetics and Formation of Co-oligomerization Products. Environ. Sci. Technol..

[47] Li J., Pang S.-Y., Wang Z., Guo Q., Duan J., Sun S., Wang L., Cao Y., Jiang J. (2021). Oxidative transformation of emerging organic contaminants by aqueous permanganate: Kinetics, products, toxicity changes, and effects of manganese products. Water Res..

[48] Zhou P., Luo M., Liu Y., Lai B., Zhang H., Ren Y., Xiong Z., He C.-S., Du Y., Zhou H. (2023). In Situ Regulation of MnO_2_ Structural Characteristics by Oxyanions to Boost Permanganate Autocatalysis for Phenol Removal. Environ. Sci. Technol..

[49] Feng X., DiSalvo F.J., Abruña H.D., Yang Y., Xiong Y. (2019). A Strategy for Increasing the Efficiency of the Oxygen Reduction Reaction in Mn-Doped Cobalt Ferrites. J. Am. Chem. Soc..

[50] Lin L.-Y., Kavadiya S., Karakocak B.B., Nie Y., Raliya R., Wang S.T., Berezin M.Y., Biswas P. (2018). ZnO_1−x_/carbon dots composite hollow spheres: Facile aerosol synthesis and superior CO_2_ photoreduction under UV, visible and near-infrared irradiation. Appl. Catal. B.

[51] Zhu L., Cheng H., Ma J., Kong Y., Qin Y., Komarneni S. (2020). Decolorization of methyl orange by MnO_2_/organic acid system: The role of Mn (III). Mater. Res. Bull..

[52] Liu X., Cui K., Yao Y., Chen X., Li C.-X., Chen Y., Hu Z., Cui M. (2024). Enhanced removal of phenolic pollutants over MnO_2_ initiated by peracetic acid: In situ generation of a heterogeneous Mn(III)-hydroperoxo complex. Chem. Eng. J..

[53] Zhao L.-X., Xiao H., Li M.-H., Xie M., Li N., Zhao R.-S. (2021). Effectively removing indole-3-butyric acid from aqueous solution with magnetic layered double hydroxide-based adsorbents. J. Hazard. Mater..

[54] Morgan J.J., Schlautman M.A., Bilinski H. (2021). Rates of abiotic MnII oxidation by O_2_: Influence of various multidentate ligands at high pH. Environ. Sci. Technol..

[55] Shao Z., Li Y., Liu C., Ai W., Luo S.-P., Liu Q. (2020). Reversible interconversion between methanol-diamine and diamide for hydrogen storage based on manganese catalyzed (de)hydrogenation. Nat. Commun..

[56] Richtrová K., Votinský J., Kalousová J., Beneš L., Zima V. (1995). Synthesis, Characterization, and Intercalation of Vanadyl Phosphate Modified with Manganese. J. Solid State Chem..

[57] Takashima T., Yamaguchi A., Hashimoto K., Irie H., Nakamura R. (2014). In situ UV-vis Absorption Spectra of Intermediate Species for Oxygen-Evolution Reaction on the Surface of MnO_2_ in Neutral and Alkaline Media. Electrochemistry.

[58] Ramanathan A., Archipov T., Maheswari R., Hanefeld U., Roduner E., Gläser R. (2008). Synthesis, Characterization and Catalytic Properties of the Novel Manganese-Containing Amorphous Mesoporous Material MnTUD-1. J. Phys. Chem. C.

[59] Klewicki J.K., Morgan J.J. (1998). Kinetic behavior of Mn (III) complexes of pyrophosphate, EDTA, and citrate. Environ. Sci. Technol..

[60] Wang X., Jones M.R., Pan Z., Lu X., Deng Y., Zhu M., Wang Z. (2024). Trivalent manganese in dissolved forms: Occurrence, speciation, reactivity and environmental geochemical impact. Water Res..

[61] Schwarzenbach G., Emeleus H.J., Sharpe A.G. (1961). The General, Selective, and Specific Formation of Complexes by Metallic Cations. Advances in Inorganic Chemistry and Radiochemistry.

[62] Law S.K. (2024). Role of Ligand Design on the Stability of Metal Complexes and Its Catalytic Properties-A Mini-Review. Biointerface Res. Appl. Chem..

[63] Wang Y., Qiu W., Lu X., Zhou X., Zhang H., Gong X., Gong B., Ma J. (2023). Nitrilotriacetic acid-assisted Mn(II) activated periodate for rapid and long-lasting degradation of carbamazepine: The importance of Mn(IV)-oxo species. Water Res..

[64] Van Aken B., Agathos S. (2002). Implication of manganese (III), oxalate, and oxygen in the degradation of nitroaromatic compounds by manganese peroxidase (MnP). Appl. Microbiol. Biotechnol..

[65] Liu Y., Zhou R., Tang Y., Li X., Xu L., Fu Y. (2024). Enhanced Mn(II)/peracetic acid by nitrilotriacetic acid to degrade organic contaminants: Role of Mn(V) and organic radicals. Sci. Rep..

[66] Chen W.-R., Liu C., Boyd S.A., Teppen B.J., Li H. (2013). Reduction of Carbadox Mediated by Reaction of Mn(III) with Oxalic Acid. Environ. Sci. Technol..

[67] Wang Y., Stone A.T. (2006). Reaction of MnIII,IV (hydr)oxides with oxalic acid, glyoxylic acid, phosphonoformic acid, and structurally-related organic compounds. Geochim. Cosmochim. Acta.

[68] Li R., Manoli K., Kim J., Feng M., Huang C.H., Sharma V.K. (2021). Peracetic Acid–Ruthenium(III) Oxidation Process for the Degradation of Micropollutants in Water. Environ. Sci. Technol..

[69] Xiong B., Wang S., Bai F., Wang J., Wang Z., Zhang L., Xie P., Wiesner M.R., Wan Y. (2019). Application of cobalt/peracetic acid to degrade sulfamethoxazole at neutral condition: Efficiency and mechanisms. Environ. Sci. Technol..

[70] Yuan D., Yang K., Pan S., Xiang Y., Tang S., Huang L., Sun M., Zhang X., Jiao T., Zhang Q. (2021). Peracetic acid enhanced electrochemical advanced oxidation for organic pollutant elimination. Sep. Purif. Technol..

[71] Shi Y., Xiao S., Qian Y., Huang C.-H., Chen J., Li N., Liu T., Zhang Y., Zhou X. (2024). Revisiting the synergistic oxidation of peracetic acid and permanganate(Ⅶ) towards micropollutants: The enhanced electron transfer mechanism of reactive manganese species. Water Res..

[72] Jiang C., Pang S., Guan C., Qiu W., Li J., Zhou Y., Yang Y., Wang Z., Jiang J., Gao Y. (2018). Is sulfate radical really generated from peroxydisulfate activated by iron(II) for environmental decontamination?. Environ. Sci. Technol..

[73] Wang J., Kim J., Ashley D.C., Sharma V.K., Huang C.-H. (2022). Peracetic Acid Enhances Micropollutant Degradation by Ferrate(VI) through Promotion of Electron Transfer Efficiency. Environ. Sci. Technol..

[74] Hu J., Xu X., Ji Y., Zhu H., Zhou Y., Zhu M., Huang Y. (2025). Role of Mn(III) intermediates in the degradation of carbamazepine via peroxymonosulfate activation by manganese single-atom catalysts: Radical and non-radical synergistic effects. Appl. Catal. B Environ. Energy.

[75] Shi W., Zhang C., Zhao H., Zhang B., Tang H., Liu Y. (2024). Picolinic acid-mediated Mn(II) activated periodate for ultrafast and selective degradation of emerging contaminants: Key role of high-valent Mn-oxo species. Water Res..

[76] Zhao X., Zhang T., Zhou Y., Liu D. (2007). Preparation of peracetic acid from hydrogen peroxide. J. Mol. Catal. A Chem..

[77] Zhao X., Cheng K., Hao J., Liu D. (2008). Preparation of peracetic acid from hydrogen peroxide, part II: Kinetics for spontaneous decomposition of peracetic acid in the liquid phase. J. Mol. Catal. A Chem..

[78] Gong W., He D., Wang X., Yan Y., Dionysiou D.D., Blaney L., Peng G. (2024). The role of Fe(IV) in the zero-valent iron biochar activated persulfate system for treatment of contaminants of emerging concern. Chem. Eng. J..

